# Can the CutiScan CS 100^®^ measure anisotropy and viscoelasticity in scar tissue after mastectomy? A reliability and validity study

**DOI:** 10.1111/srt.13120

**Published:** 2021-11-09

**Authors:** Mieke Anthonissen, Eric Van den Kerckhove, Peter Moortgat, Inge Geraerts, Nele Devoogdt, Tessa De Vrieze, An De Groef

**Affiliations:** ^1^ Scar After‐Care & Research Oscare‐Organization for Burns Antwerp Belgium; ^2^ Department of Rehabilitation Sciences KU Leuven‐University of Leuven Leuven Belgium; ^3^ Department of Rehabilitation Sciences and Physiotherapy MOVANT University of Antwerp Antwerp Belgium; ^4^ Department of Physical Medicine and Rehabilitation UZ Leuven Leuven Belgium; ^5^ Department of Plastic Surgery AZ Maastricht Maastricht The Netherlands; ^6^ Department of Vascular Surgery and Department of Physical Medicine and Rehabilitation Centre for Lymphedema University Hospitals Leuven Leuven Belgium; ^7^ Improving Care in Edema and Oncology International Research Group; ^8^ Pain in Motion International Research Group

**Keywords:** anisotropy, breast cancer, CutiScan CS 100^®^, mastectomy, reliability, scar adhesions, validity, viscoelasticity

## Abstract

**Background:**

Scars have different biomechanical characteristics, including anisotropy and viscoelasticity compared to healthy skin. To assess these characteristics, the CutiScan CS 100^®^ can be used. The aim of the present study is to investigate reliability and validity of this device in breast cancer patients.

**Materials and methods:**

Thirty female patients, with scar adhesions following mastectomy were assessed with the CutiScan CS 100^®^. Maximal distensibility (pixels) (V1), after‐suction return rate (pixels) (V2), and their ratio (%) (V3) at three points on and around the scar were assessed as measures of viscoelasticity. For intra‐ and interrater reliabilities, the intra‐class correlation coefficient (ICC) and its 95% confidence intervals were calculated. The standard error of measurement (SEM) was calculated to interpret reproducibility of these measurements. To investigate criterion validity of the measurement of anisotropy, measurements in the direction of healthy skin were compared with measurements in the direction of the scar, using a paired *t*‐test.

**Results:**

V1, V2, and V3 show poor to moderate intrarater reliability (ICC 0.00–0.72) and interrater reliability (ICC 0.00–0.53). The maximum displacement (V1) on the measurement point above the scar shows the best reliability (ICC 0.33–0.72). The SEM is about the same for all parameters at all three points. The paired sample *t*‐test showed a significant difference (*p* < 0.05) between V1 in the direction towards the scar versus the measurement towards healthy tissue, on the point below the scar.

**Conclusion:**

These first reliability and validity results of the CutiScan CS 100^®^ for measuring anisotropy and viscoelasticity in scar tissue adhesions after mastectomy seem promising. Further research is needed addressing the limitations of the present study design.

## 1 INTRODUCTION

Breast cancer is the second most common cancer overall with 1.7 million cases or 11.9% of all cancer cases worldwide.^1^ Survival rate continues to improve over time due to early screening procedures and the development of new treatments.^1^ However, many patients still suffer from adverse effects because of these treatments.^2^

Local treatment modalities including radiotherapy and surgery may result in specific side effects involving the soft tissues at the upper limb region.^2^ At the level of the trunk, surgery, in particular mastectomy, may lead to scar tissue adhesions.^3^ Radiotherapy‐induced fibrosis is a known long‐term side effect resulting in a multitude of symptoms.^4^ Both scar tissue adhesions and fibrosis may contribute to shoulder movement restrictions, lymphoedema, pain, and a decrease in a patient's functional quality of life by esthetical, psychological, and physical complaints.^5^, ^6^, ^7^, ^8^

Scar tissue formation involves alterations in two important components of the extracellular matrix: properties of collagen (which contributes to the elasticity and strength) and elastin (which contributes to the structure of the skin and the ability to recoil).^9^, ^10^ The alterations in collagen induce a higher orientation index, which indicates that the organization of collagen in scar tissue is more parallel aligned. By contrast, normal skin has a random organization.^9^, ^10^ The collagen bundles in scar tissue are also thinner and the distance between the bundles decreases.^9^, ^11^ The distribution and features of elastin are altered in the way that the interlacing network evolves towards a more homogeneous distribution. The fiber thickness decreases in both superficial and deep dermis. However, similar to normal skin, the fibers in the deep dermis remain thicker than in the superficial dermis.^11^, ^12^

Regarding the quality and functionality of scare tissue, two important biomechanical characteristics should be considered. First, for anisotropy (i.e., directional variations in tissue organization) an important difference between scar tissue and normal skin had been reported.^9^, ^10^, ^11^, ^13^ Scar tissue has a lower rate of anisotropy because collagen and elastin are more parallel aligned.^9^, ^10^, ^11^, ^13^ Second, viscoelasticity also differs between scar tissue and healthy skin. There is a lower degree of viscoelasticity in scar tissue due to the smaller collagen and thinner elastin bundles on the one hand, and because of an adaptation in the presence of proteoglycan on the other hand. This leads to a stiffer and more viscous behavior, and therefore causes a faster relaxation of the tissue after being extended.^14^

Proper evaluation of anisotropy and viscoelasticity is warranted to understand the severity of adhesions after surgery for breast cancer, given their impact on the quality of life of patients.^5^, ^6^, ^7^, ^8^ Preferably, objective assessment methods are used, in combination with self‐reported outcome measure to have a comprehensive understanding of mastectomy scars and their impact.^15^, ^16^ Skin anisotropy and viscoelasticity can be measured with the CutiScan CS 100^®^. This device uses mechanical force in combination with imaging.^17^ The CutiScan CS 100^®^ has already been used to compare the changes in the biomechanical properties of the healthy human skin (viscoelasticity and anisotropy) depending on the anatomical site,^18^, ^19^ to investigate the influence of hydration on the biomechanical behavior of the human skin,^20^ and to evaluate anisotropic properties of striae distensae.^21^ However, to our knowledge, it is not certain whether viscoelasticity and anisotropy measured with the CutiScan CS 100^®^ are reliable and valid outcome measures for mastectomy scars. Therefore, the aim of the current study is to investigate intrarater reliability, interrater reliability, and criterion validity of the CutiScan CS 100^®^ in breast cancer patients with scar tissue adhesions after a mastectomy procedure.

## 2 MATERIALS AND METHODS

This study was approved by the Ethical Committee of the University Hospitals of Leuven (s54579). All participants gave a written informed consent.

### 2.1 Participants

A sample of 30 women treated for breast cancer was recruited at the Multidisciplinary Breast Center of the University Hospitals of Leuven in January and February 2015. The inclusion criteria were a fully healed mastectomy scar (i.e., full wound closure after removal of stitches/sutures) of a unilateral radical mastectomy as treatment for breast cancer. Other (neo‐)adjuvant treatment modalities, including chemotherapy, radiotherapy, targeted therapy, and/or endocrine therapy were either finished or ongoing. Patients with complications such as lymphedema (determined with circumference measurements for the arm and visual inspection and palpation for the trunk) and/or seroma at the level of the trunk were excluded.

### 2.2 Patient characteristics

The following patient characteristics were collected through self‐report: age, body mass index (BMI), time after surgery, and previous or ongoing (neo‐)adjuvant cancer therapy modalities.

### 2.3 Procedure

**FIGURE 1:**
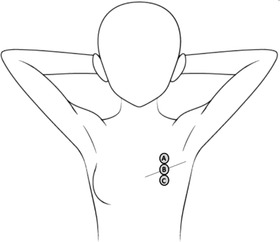
Schematic overview of the predetermined position and measurement points

During the measurement, the subjects were instructed to lie down in a predetermined position with the arms behind the head (Figure 1). In case of pain or discomfort, the subject was allowed to place the arms alongside the body during the whole duration of the measurement.

Three measuring points were defined, including a point 2 cm above the scar (point A), a point at the center of the scar (point B), and a point 2 cm below the center of the scar (point C) (Figure 3). The exact center point of the scar (point B) was determined by using a measuring tape. Next, a line perpendicular to the direction of the scar was drawn to determine points A and C. At each point, a circle was drawn with the use of a circular template of the probe of the CutiScan CS 100^®^ device. The first rater (A.D.G.) held a master's degree in rehabilitation sciences and 3 years of clinical experience in treatment of myofascial dysfunctions in breast cancer patients. The second rater was a master's student in rehabilitation sciences without clinical expertise. Both raters received the same 2‐h training with the CutiScan CS 100^®^ device.

To investigate the intrarater reliability, the same observer (A.D.G.) conducted two consecutive measurements, with 3 min pause in between. Three minutes after the second recording of the first observer (A.D.G.), a second observer (student) conducted the same site to investigate the interrater reliability.

### 2.4 CutiScan CS 100^®^

The CutiScan CS 100^®^ from Courage + Khazaka electronic GmbH, consists of a probe (diameter of 14 mm) with a suction ring (inside diameter of 5 mm) and a high‐resolution Charge‐Coupled Device (CCD)‐camera inside the probe. The device combines the assessment of mechanical properties of the skin with imaging.^17^

**FIGURE 2:**
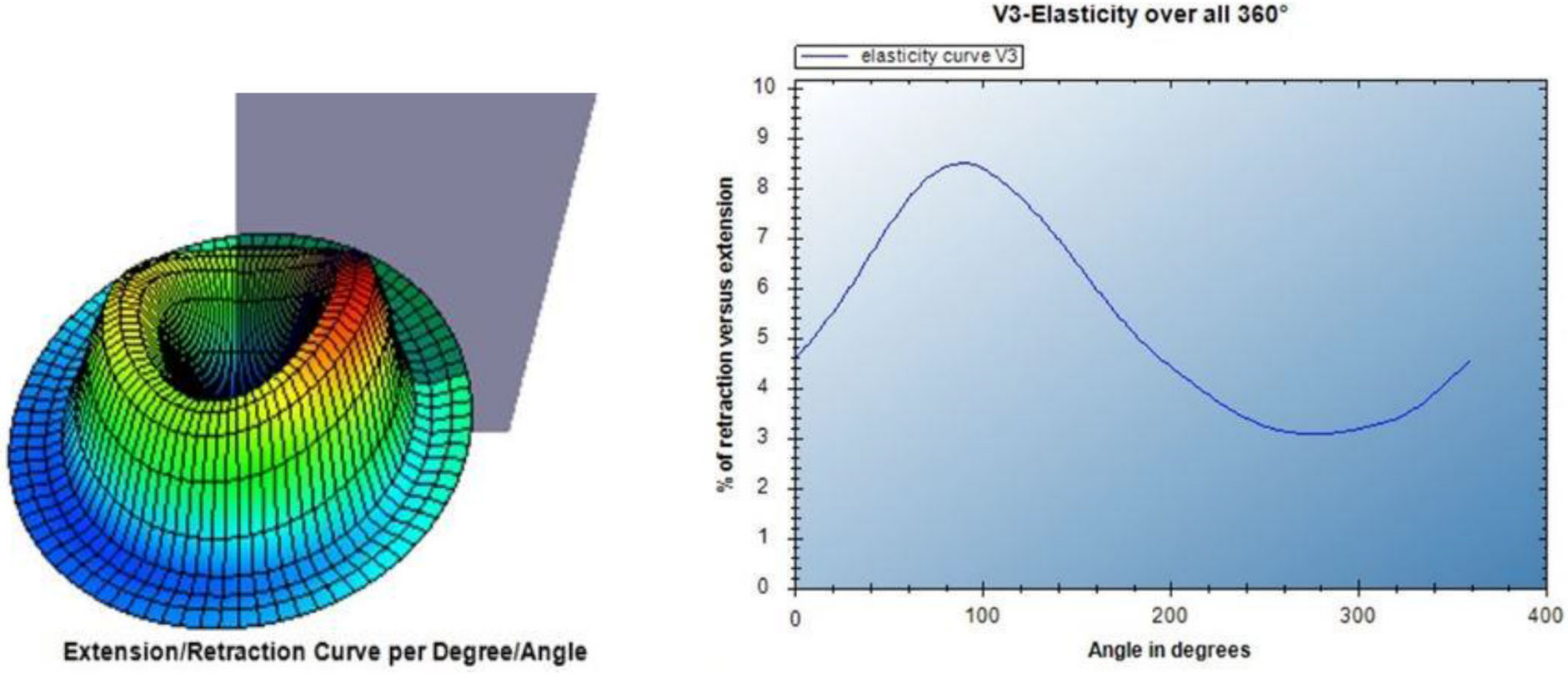
Left: Displacement of each pixel in a 3D illustration; right: elasticity curve (V3) overall 360° (figures derived from Courage + Khazaka electronic GmbH)^17^

During a measurement, the probe is placed perpendicular on the skin for a 3‐s period of suction, in which a constant pressure of 400 mbar is applied. This is followed by a relaxation period of another 3 s. During the suction and relaxation period, the camera inside the probe records the displacement of the skin in a 360° angle. The high‐resolution CCD‐camera monitors the displacement of each pixel in video format, by using an optical flow algorithm. An optical flow algorithm is a pattern of movement of the skin during the suction and relaxation phase, set in a visual scene (Figure 2).

**FIGURE 3:**
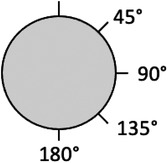
Cross‐section of the probe with the angles used for analyses

From this video, graphs are generated which in turn provide the following measurement parameters for each 360° angle (*x*‐axis): (1) maximum displacement during the suction time (pixels) (V1), (2) the rate of skin returning after suction (pixels) (V2), and (3) the ratio between V1 and V2 (V3) calculated as a percentage, showing elasticity of the skin (%). An example of a graph for elasticity (V3) for each angle is given in Figure 3.

For the purpose of the present study, the three parameters (V1–V3) at 45°, 90°, 135°, and 180° were used for analyses (Figure 3). In addition, V1–V3 of the sum of all angles (360°) were used for analyses as well.

### 2.5 Statistical analysis

For the intra‐ and interrater reliabilities, the intra‐class correlation coefficient (ICC 2,1) and its 95% confidence intervals (CI) were calculated from the parameters V1 (maximum displacement during the suction time in pixels), V2 (after‐suction return rate in pixels), and V3 calculated as a percentage (the ratio between V1 and V2) in four directions (45°, 90°, 135°, and 180°) and for the total sum of the 360°. According to the guidelines provided by Portney,^22^ an ICC below 0.50 indicates a poor reliability ), between 0.51 and 0.79 a moderate reliability, between 0.80 and 0.90 a good reliability, and above 0.90 an excellent reliability. The standard error of measurement (SEM) was calculated to interpret the amount of errors in a measurement, and therefore to interpret reproducibility. The SEM provides information whether the measured change is due to the measurement error or due to the real change. The following formula was used: $\mathrm{SEM}=SD_{\left(r1r2\right)}\sqrt{1-\mathrm{ICC}}$, where SD_(r1r2)_ indicates the average standard deviation of the two ratings.

To investigate the criterion validity of the CutiScan CS 100^®^ for the measurement of anisotropy, the V1–V3 in the direction of the healthy skin were compared with the results in the direction of the scar on both measurement points A and C. For point A, this means 5° versus 135° and for point C, this means 45° versus 180° as these were the data points available the furthest apart on the respective measurement points. Point B (on the scar) was not considered for criterion validity as no distinction between healthy and scar tissue can be made on this point. Given that half of the outcome parameters showed normal distribution (based on the Shapiro–Wilk test), a powerful parametric paired sample *t*‐test was chosen for validity testing. Statistical Package for the Social Sciences (SPSS) IBM Corp, Released 2019, IBM SPSS Statistics for Macintosh, Version 26.0, IBM Corp, Armonk, NY was used for analyses.

## 3 RESULTS

Thirty female patients with a mean (SD) age of 53.9 (10.9) years and a mean BMI (SD) of 25.2 (4.0) kg/m^2^ were included. The mean (SD) time after surgery was 1.2 (0.5) years. Of all 30 included patients: 13 women (43%) underwent chemotherapy of which 11 (37%) (neo‐)adjuvant chemotherapy, 30 women (100%) received radiotherapy, 24 (80%) endocrine therapy, and 5 (14%) received targeted therapy.

Tables 1, 2, 3 give an overview of the maximum displacement during suction time (V1), after‐suction return rate (V2), and their ratio (V3) for measurement points A–C, respectively.

**TABLE 1:** Overview of reliability of the maximum displacement (V1), suction return rate (V2), and their ratio (V3) for the measurement at the point above the scar (point A)

	V1, mean (SD) (pixel)	V1, intrarater ICC (95% CI)	V1, interrater ICC (95% CI)	SEM (95% CI)	V2, mean (SD) (pixel)	V2, intrarater ICC (95% CI)	V2, interrater ICC (95% CI)	SEM (95% CI)	V3, mean (SD) (%)	V3, intrarater ICC (95% CI)	V3, interrater ICC (95% CI)	SEM (95% CI)
45°	78.20 (23.22)	**0.44** (0.10 to 0.69)	**0.33** (−0.03 to 0.61)	19.01 (−18.24 to 56.27)	8.00 (9.59)	**0.19** (−0.18 to 0.51)	**0.00** (. to .)	9.59 (−9.21 to 28.39)	10.39 (12.12)	**0.06** (−0.30 to 0.41)	**0.00** (. to .)	12.12 (−11.64 to 35.88)
90°	74.80 (23.49)	**0.46** (0.12 to 0.70)	*0.52* (0.21 to 0.74)	16.27 (−15.62 to 48.16)	7.08 (8.45)	**0.08** (−0.29 to 0.42)	**0.09** (−0.27 to 0.43)	8.06 (−7.74 to 23.86)	10.46 (13.44)	**0.03** (−0.33 to 0.38)	**0.15** (−0.22 to 0.48)	12.39 (−11.89 to 36.67)
135°	76.18 (26.65)	*0.72* (0.49 to 0.86)	*0.53* (0.22 to 0.75)	18.27 (−17.54 to 54.08)	8.54 (12.98)	**0.00** (. to .)	**0.09** (−0.27 to 0.43)	12.38 (−11.88 to 36.64)	11.93 (17.92)	**0.00** (. to .)	**0.26** (−0.10 to 0.57)	15.42 (−14.80 to 45.64)
180°	78.50 (25.45)	*0.64* (0.37 to 0.81)	**0.42** (0.08 to 0.68)	19.38 (−18.60 to 57.36)	9.96 (15.12)	**0.00** (. to .)	**0.04** (−0.32 to 0.39)	14.81 (−14.22 to 43.84)	14.08 (20.33)	**0.02** (−0.34 to 0.37)	**0.28** (−0.09 to 0.58)	17.25 (−16.56 to 51.06)
Sum	27 547.91 (6845.97)	*0.51* (0.19 to 0.73)	**0.39** (0.04 to 0.66)	5346.87 (−5132.99 to 15 826.74)	3155.00 (3178.09)	**0.19** (−0.18 to 0.51)	**0.09** (−0.28 to 0.43)	3031.70 (−2910.43 to 8973.83)	4272.66 (4224.65)	**0.15** (−0.21 to 0.48)	**0.11** (−0.26 to 0.45)	3985.53 (−3826.11 to 11 797.17)

*Note*: Bold values indicate poor reliability (ICC < 0.50), italic values indicate moderate reliability (ICC 0.50–0.80). (. to .) means that SPSS was not able to show these results because of estimation problems.

Abbreviations: CI, confidence interval; ICC, intra‐class correlation coefficient; SD, standard deviation; SEM, standard error of measurement.

**TABLE 2:** Overview of reliability of the maximum displacement (V1), suction return rate (V2), and their ratio (V3) for the measurement at the point on the scar (point B)

	V1, mean (SD) (pixel)	V1, intrarater ICC (95% CI)	V1, interrater ICC (95% CI)	SEM (95% CI)	V2, mean (SD) (pixel)	V2, intrarater ICC (95% CI)	V2, interrater ICC (95% CI)	SEM (95% CI)	V3, mean (SD) (%)	V3, intrarater ICC (95% CI)	V3, interrater ICC (95% CI)	SEM (95% CI)
45°	87.75 (28.93)	*0.56* (0.25 to 0.76)	*0.51* (0.19 to 0.73)	20.25 (−19.44 to 59.94)	19.63 (17.42)	**0.22** (−0.15 to 0.53)	**0.05** (−0.32 to 0.39)	16.98 (−16.30 to 50.26)	23.40 (20.11)	**0.29** (−0.07 to 0.58)	**0.00** (. to .)	20.11 (−19.31 to 59.53)
90°	86.84 (30.41)	*0.60* (0.31 to 0.79)	*0.59* (0.30 to 0.78)	19.47 (−18.69 to 57.63)	22.48 (16.94)	**0.02** (−0.34 to 0.37)	**0.10** (−0.27 to 0.44)	16.07 (−15.42 to 47.57)	27.10 (20.51)	**0.15** (−0.22 to 0.48)	**0.10** (−0.26 to 0.44)	19.46 (−18.68 to 57.60)
135°	85.07 (30.69)	**0.44** (0.10 to 0.69)	*0.54* (0.22 to 0.75)	20.81 (−19.98 to 61.60)	24.09 (18.49)	**0.00** (. to .)	**0.09** (−0.28 to 0.43)	17.64 (−16.93 to 52.21)	29.19 (21.25)	**0.16** (−0.21 to 0.48)	**0.07** (−0.29 to 0.42)	20.49 (−19.67 to 60.65)
180°	84.69 (30.25)	**0.29** (−0.07 to 0.59)	**0.46** (0.13 to 0.70)	22.23 (−21.34 to 65.80)	25.15 (20.93)	**0.00** (. to .)	**0.21** (−0.15 to 0.53)	18.60 (−17.86 to 55.06)	29.91 (20.96)	**0.14** (−0.23 to 0.47)	**0.20** (−0.17 to 0.52)	18.75 (−18.00 to 55.50)
Sum	31 085.32 (8632.16)	*0.60* (0.30 to 0.78)	*0.57* (0.27 to 0.77)	5660.49 (−5434.07 to 16 755.05)	8335.81 (4968.78)	**0.32** (−0.04 to 0.61)	**0.46** (0.12 to 0.70)	3651.29 (−3505.23 to 10 807.82)	9616.95 (5790.64)	**0.40** (0.05 to 0.66)	**0.15** (−0.21 to 0.48)	5338.71 (−5125.17 to 15 802.58)

*Note*: Bold values indicate poor reliability (ICC < 0.5), italic alues indicate moderate reliability (ICC 0.5–0.8). (. to .) means that SPSS was not able to show these results because of estimation problems.

Abbreviations: CI, confidence interval; ICC, intra‐class correlation coefficient; SD, standard deviation; SEM, standard error of measurement.

**TABLE 3:** Overview of reliability of the maximum displacement (V1), suction return rate (V2), and their ratio (V3) for the measurement at the point below the scar (point C)

	V1, mean (SD) (pixel)	V1, intrarater ICC (95% CI)	V1, interrater ICC (95% CI)	SEM (95% CI)	V2, mean (SD) (pixel)	V2, intrarater ICC (95% CI)	V2, interrater ICC (95% CI)	SEM (95% CI)	V3, mean (SD) (%)	V3, intrarater ICC (95% CI)	V3, interrater ICC (95% CI)	SEM (95% CI)
45°	69.56 (24.43)	*0.60* (0.31 to 0.79)	*0.50* (0.17 to 0.72)	17.27 (−16.58 to 51.12)	16.49 (14.41)	**0.16** (−0.21 to 0.49)	**0.01** (−0.35 to 0.36)	14.34 (−13.77 to 42.45)	25.08 (20.51)	**0.31** (−0.05 to 0.60)	**0.00** (. to .)	20.51 (−19.69 to 60.71)
90°	70.65 (24.57)	*0.64* (0.36 to 0.81)	**0.48** (0.15 to 0.72)	17.72 (−17.01 to 52.45)	19.13 (14.09)	**0.12** (−0.25 to 0.46)	**0.26** (−0.11 to 0.56)	12.12 (−11.64 to 35.88)	29.61 (21.72)	**0.31** (−0.06 to 0.60)	**0.35** (−0.01 to 0.63)	17.51(−16.81 to 51.83)
135°	65.10 (23.07)	*0.55* (0.24 to 0.75)	*0.50* (0.18 to 0.73)	16.31 (−15.66 to 48.28)	15.84 (13.61)	**0.27** (−0.10 to 0.57)	**0.22** (−0.15 to 0.53)	12.02 (−11.54 to 35.58)	26.76 (23.15)	**0.34** (−0.02 to 0.62)	**0.29** (−0.07 to 0.58)	19.51 (−18.73 to 57.75)
180°	60.49 (21.69)	**0.34** (−0.02 to 0.62)	**0.31** (−0.05 to 0.60)	18.02 (−17.30 to 53.34)	11.20 (11.59)	**0.49** (0.17 to 0.72)	**0.30** (−0.06 to 0.59)	9.70 (−9.31 to 28.71)	21.23 (21.11)	*0.61* (0.33 to 0.79)	**0.40** (0.05 to 0.66)	16.35 (−15.70 to 48.40)
Sum	23 276.70 (6782.32)	**0.49** (0.16 to 0.72)	**0.49** (0.17 to 0.72)	4843.55 (−4649.81 to 14 336.91)	4496.67 (3298.27)	**0.22** (−0.15 to 0.53)	**0.00** (. to .)	3298.27 (−3166.34 to 9762.88)	7748.94 (5656.21)	**0.42** (0.07 to 0.67)	**0.11** (−0.25 to 0.45)	5336.06 (−5122.62 to 15 794.74)

*Note*: Bold values indicate poor reliability (ICC < 0.5), italic values indicate moderate reliability (ICC 0.5–0.8). (. to .) means that SPSS was not able to show these results because of estimation problems.

Abbreviations: CI, confidence interval; ICC, intra‐class correlation coefficient; SD, standard deviation; SEM, standard error of measurement.

For maximum displacement (V1), the results show that there is, depending on the angle, a poor (ICC 0.29–0.49) to moderate (ICC 0.51–0.72) intrarater reliability. Also, for the interrater reliability, the results show a poor (ICC 0.31–0.49) to moderate (ICC 0.50–0.59) reliability. The measurement point that shows the highest reliability values for maximum displacement (V1) is the point above the scar (point A). The SEM (16.27–3651.29) is about the same for all three points of measurement. For the after‐suction return rate (V2), there is a poor reliability for both intrarater reliability (ICC 0.00–0.49) and interrater reliability (ICC 0.00–0.46) for all measurement points. The SEM (16.27–5660.49) is about the same for all three points of measurement. The ratio of the previous parameters (V3) shows poor intra‐ and interrater reliabilities at all points as well.

Table 4 shows the paired sample *t*‐test for the criterion validity of the CutiScan CS 100^®^. There is a significant difference (*p* < 0.05) between the measurement of V1 in the direction towards the scar (i.e., at 45°) versus the measurement towards healthy tissue (i.e., at 180°) on point C (below the scar). This result indicates a good criterion validity of anisotropy at this point. No similar results were found for point A (above the scare).

**TABLE 4:** Paired sample *t*‐test

	*p*‐Value	Mean (SD) 5°	Mean (SD) 135°
Measurement point A above the scar (5° vs. 135°)			
V1	0.419	80.95 (23.58)	76.18 (27.10)
V2	0.678	9.98 (13.90)	8.54 (13.20)
V3	0.884	11.30 (15.61)	11.93 (18.23)
Measurement point C below the scar (45° vs. 180°)			
	*p*‐Value	Mean (SD) 45°	Mean (SD) 180°
V1	0.015^*^	69.56 (24.43)	60.49 (21.69)
V2	0.091	16.49 (14.41)	11.20 (11.59)
V3	0.413	25.08 (20.51)	21.23 (21.11)

Abbreviation: SD, standard deviation.^*^Significance (*p* < 0.05).

## 4 DISCUSSION

It has been suggested that skin anisotropy and viscoelasticity can be measured with the CutiScan CS 100^®^. This device has already been used to compare the changes in the biomechanical properties of healthy human skin in different ages and at different anatomical sites. Also, the influence of hydration on the biomechanical behavior of human skin has already been investigated with the CutiScan CS 100^®^.^18^, ^19^, ^20^ However, the reliability and validity of the CutiScan CS 100^®^ for the assessment of viscoelasticity and anisotropy in non‐healthy skin were not yet established. Therefore, the aim of the present study is to investigate the intra‐ and interrater reliabilities and the criterion validity (for anisotropy) of the CutiScan CS 100^®^ in breast cancer patients with scar tissue adhesions after mastectomy.

Results show that the maximal displacement (V1) is more reliable than the return rate (V2). The maximal displacement (V1) shows the best reliability at the measurement point on the scar itself. The intrarater reliability seems higher than the interrater reliability. However, overall, we have to conclude that the reliability is poor for the assessment of mastectomy scars in breast cancer patients. Furthermore, results indicated a good criterion validity of anisotropy only at the measurement point below the scar.

Several explanations for the poor reliability can be postulated. First, the amount of pressure used on the probe by the raters was not standardized. The influence of this limitation has already been explored in a previous study with the Cutometer^®^.^23^ They found that varying the pressure on the probe alters the skin elasticity measures, with in particular a decrease in elasticity measures when applying more pressure.^25^ Second, the measurement on the scar showed the highest reliability probably because of higher variability in measurements at this measurement point. At last, it should be noted that the inside diameter of the suction ring is only 5 mm and therefore too small to measure the whole scar. Small deviations in assessment location, despite using a mark on the skin, may contribute to the poor reliability.

To investigate the criterion validity for anisotropy, measurements by the CutiScan CS 100^®^ towards scar tissue versus towards healthy skin were compared. Analysis of the results indicated that only maximal displacement (V1) of the measurement point below the scar (point C) reached the significance level. We would expect a similar result in the measurement point above the scar. An explanation why no directionality could be measured above the scar is not clear. Although, limitations in the assessment method contributing to the poor reliability as described above may also cause limitations in the evaluation of the validity. In previous research, the CutiScan CS 100^®^ was able to demonstrate that striae distensae are more anisotropic compared to adjacent normal skin.^21^ Kim et al.^21^ compared biomechanical properties over the total 360° in a body region with striae distensae versus an adjacent body region with normal skin, instead of one single angle (5° vs. 135° and 45° vs. 180°) at points above and below the mastectomy scar in our study. This different approach may be more suitable to evaluate anisotropy and thus criterion validity and should therefore be considered as a limitation of the present study. It should also be noted that characteristics of striae distensae are remarkably different from characteristics of mastectomy scars, radiotherapy‐related fibrosis, and related adhesions (e.g., etiological mechanism), making a direct comparison of the result difficult.

Besides the poor reliability and validity, other findings have to be discussed. First, we would expect scar tissue to return faster to its original state during the relaxation period because of a change in its viscoelastic behavior. Scar tissue has an altered elastin and collagen structure and an increased amount of proteoglycans. This leads to a stiffer and more viscous behavior and causes faster relaxation of the tissue after extension.^9^ Data of the present study indicate that this expected behavior did not occur. On the contrary, we occasionally encountered a negative value for the after‐suction return rate (V2). The negative V2 values potentially indicated that scar tissue extends even further after being released from suction, where scar tissue is normally expected to have a fast relaxation, resulting in a large and positive value after suction time (V2). The most plausible explanation for these results is that scarred skin has an irregular and thicker surface and might get stuck more easily in the camera opening frame. Applying the probe with harder pressure can cause the scar to get caught during relaxation time and would produce negative values. Second, since we were especially interested in scar adhesions, we did not take into account other scar‐related characteristics such as scar color or thickness. Since patients were measured 1.2 ± 0.5 years after surgery all scars were fully healed making these parameters relevant. However, it should be noted that scars may still be in an active maturation phase. Additionally, BMI and related fat distribution at the assessment location may have influenced the results, particularly in this sample with borderline overweight since subcutaneous adipose tissue is known to influence skin elasticity.^24^ While the effects of estrogen on skin hydration, elasticity, and thickness have been widely studied,^25^ to which extent hormone therapy for breast cancer alters skin properties and thus possibly has impacted are results needs to be investigated as well.

### 4.1 Clinical implications

We aimed to investigate the reliability and validity of the CutiScan CS 100^®^ in measuring scar tissue of patients who had undergone a mastectomy procedure for breast cancer. The results of this study indicate that the CutiScan CS 100^®^ is not a suitable device to measure anisotropy in this population for scientific purposes. However, the visual representation of the CutiScan CS 100^®^, with 3D illustration of the displacement of each pixel (Figure 2) can be useful in clinical practice.

### 4.2 Strengths and limitations

As described above, the lack of standardization in the applied pressure on the probe may have resulted in irregular measurements. However, the amount of pressure applied on the probe was exercised during different training sessions, before the start of the trial. Also, a double‐sided sticking ring to attach the probe to the skin may add in the standardization of the measurement as well. Also, in case of pain or discomfort, patients were allowed to place their arms alongside their body during the measurement. This difference in position, and thus tension on the skin, was not considered in the analyses. The strength of this study was to exclude 0° from analysis for both reliability and criterion validity, and instead to start from 5°. Because the time to get to full suction power after placing the probe on the skin, the first degrees may show errors.

### 4.3 Further research

To achieve more regular and reproducible measurements, use of a double‐sided sticking ring should be investigated to fix the probe on the skin and to avoid the skin getting stuck in the camera opening frame, and not applying any pressure on the probe during application. Adjustment of the probe may be considered to avoid application of high pressure on the probe. To improve reliability, we suggest to perform at least three repetitive measurements per location and calculate the average of these measurements. Another tool that has been used in the past to measure anisotropy, the Reviscometer^®^, was found to be a reliable measurement tool on scar tissue.^13^ Further studies are needed to compare the reliability and validity of the CutiScan CS 100^®^ and the Reviscometer^®^ for the evaluation of scar tissue in different populations.

## 5 CONCLUSION

Although intra‐ and interrater reliabilities and validity of the CutiScan CS 100^®^ for the assessment of viscoelasticity and anisotropy of scar tissue after a mastectomy procedure for breast cancer was not strongly established, these first results are promising. Further research should address the limitations in the current study design and explore if standardization of pressure on the probe, the use of a double‐sided sticking ring, increasing the diameter of the suction opening, and adding repetitive measurements per location can increase reliability and validity in the breast cancer population.
